# Clinical analysis of ultrasound-guided radiofrequency ablation for recurrent colorectal liver metastases after hepatectomy

**DOI:** 10.1186/s12957-020-01849-0

**Published:** 2020-04-20

**Authors:** Xiao-xiang Fan, Shu-yi Lv, Mei-wu Zhang, Xiao-yu Dai, Jian-pei Zhao, Da-feng Mao, Yan Zhang

**Affiliations:** 1 Department of Interventional Therapy, Hwa Mei Hospital, University of Chinese Academy of Sciences, Ningbo, People’s Republic of China; 2 Ningbo Institute of Life and Health Industry, University of Chinese Academy of Sciences, Ningbo, People’s Republic of China; 3 Key Laboratory of Diagnosis and Treatment of Digestive System Tumors of Zhejiang Province, Ningbo, 315010 Zhejiang People’s Republic of China; 4 Department of Colorectal surgery, Hwa Mei Hospital, University of Chinese Academy of Sciences, Ningbo, People’s Republic of China

**Keywords:** Radiofrequency ablation, Repeat hepatic resection, Ultrasound-guided, Liver metastases, Recurrence rate, Clinical outcome

## Abstract

**Background:**

RFA is designed to produce localized tumor destruction by heating the tumor and surrounding liver tissue, especially suitable for patients who do not qualify for hepatic resection. Many studies have reported that RFA was inferior to hepatectomy in the treatment of recurrent colorectal liver metastases. However, strong evidence is lacking in the literature. This study aimed to investigate the effect and clinical outcome of percutaneous ultrasound-guided RFA and repeat hepatic resection for recurrent colorectal liver metastases after hepatectomy.

**Methods:**

From January 2007 to January 2014, 194 patients with recurrent colorectal liver metastases after hepatectomy diagnosed in our hospital was performed, and then divided into two groups based on different regimens: repeat hepatic resection group and RFA group. The clinical data of the two groups were analyzed. After treatment, the liver function-related indexes, complication rate, survival rate, and tumor recurrence of the two groups were recorded. The difference in short-term and long-term effects between repeat hepatic resection and RFA was identified by propensity score analysis.

**Results:**

The number of metastases and the proportion of left and right lobe involved by tumor and preoperative chemotherapy in the RFA group were higher than those in the repeat hepatic resection group. The clinical data showed no significant difference between the two groups after using propensity score analysis. Compared with the RFA group, the liver function of the repeat hepatic resection group was significantly improved. After adjustment for potential confounders, no significant difference in liver function-related indexes was found between RFA and repeat hepatic resection, and the incidence of complications in the RFA group was lower. In survival analysis, there was no significant difference in OS and DFS between the two groups.

**Conclusions:**

RFA is a safe and effective therapeutic option for patients with recurrent colorectal liver metastases after hepatectomy.

## Background

Colorectal cancer is one of the most common malignant tumors and has high morbidity and mortality. As the hub of the portal and vena cava systems, the liver is the primary organ involved in the invasion of colorectal cancer [1–5]. The latest statistics show that 20% to 25% of patients have liver metastases at the time of the initial diagnosis, and about 50% of colorectal cancer patients will develop liver metastasis during the course of their diseases [6]. Although the 5-year survival rate of patients with liver metastases from colorectal cancer can significantly be improved by hepatectomy, the high postoperative recurrence has become one of the main reasons that hinder patients from the long-term survival [7–9]. Several studies have found that more than half of patients relapsed within 2 years after initial surgery, and new metastatic foci appeared in 50% to 70% of patients [10, 11]. For these patients, repeat hepatic resection is the standard and effective treatment regimen and associated with few complications and low mortality [12, 13]. However, repeat hepatic resection is technically demanding because of proximity to vital structures and is less effective for those patients with multiple lesions, large metastatic foci, and short disease-free interval. Hence, non-surgical treatment should be considered [14–18]. Thermal ablation procedures such as radiofrequency ablation (RFA) offers the advantages of security, efficiency, minimally invasive, and can cause coagulation necrosis of the tumor with avoidance of unnecessary surgery, especially for patients who are not candidates for hepatic resection [19, 20]. For this reason, RFA has been widely applied in clinical treatment [21, 22]. Relevant studies have suggested that the limitation of RFA is an increased risk of local tumor recurrence, and the expected effect may not be achieved [23, 24]. In essence, there is little study on the treatment of patients with recurrent colorectal liver metastases after resection, and the clinical efficacy and prognostic impact of RFA for this disease are still controversial. Therefore, this study explored the role and status of percutaneous ultrasound-guided RFA in the comprehensive treatment of recurrent colorectal liver metastases after hepatectomy.

## Methods

### Study population and data collection

Patients who underwent hepatectomy for colorectal cancer liver metastases at Hwa Mei hospital between January 2007 and January 2014 were enrolled. Inclusion criteria include (1) all patients with liver metastases from colorectal cancer that had already undergone radical resection, and (2) the patient was diagnosed as intrahepatic recurrence by B-ultrasound, CT, or MRI and serum tumor markers. A total of 194 patients met the inclusion criteria (74 females, 120 males; mean age 60.5 years, range 37–83 years). Indications for repeat hepatic resection included (1) complete resection (R0) of intrahepatic lesions can be achieved, (2) patients have enough capacity of the residual liver (> 30%), (3) with normal hepatic inflow and outflow tract, and (4) without surgical contraindication.

Indications for RFA are as follows: (1) the number of tumors ≤ 3 and the maximum diameter of the tumor ≤ 5 cm, or the number of tumors > 4 and the maximum diameter of the tumor ≤ 3 cm; (2) patients have the puncture path for percutaneous ablation; (3) R0 ablation can be achieved; (4) platelets ≥ 60 × 10^9^/L and prothrombin activity ≥ 50%. According to the above criteria, patients were divided into repeat hepatic resection (50 cases) and the RFA group (144 cases).

### RFA

Among the 144 cases, 90 had primary colon cancer, 54 had rectal cancer, and six with extrahepatic metastasis. Primary partial hepatectomy was performed in 132 patients and repeated partial hepatectomy in 12 patients. Fifty-seven cases of hepatic metastases involved a single lobe and 87 cases of the double lobe. Sixty-nine cases were single metastatic lesions and 75 cases were multiple lesions. The time for new intrahepatic neoplasia detection was (6.5 + 8.0) months.

**Fig. 1:**
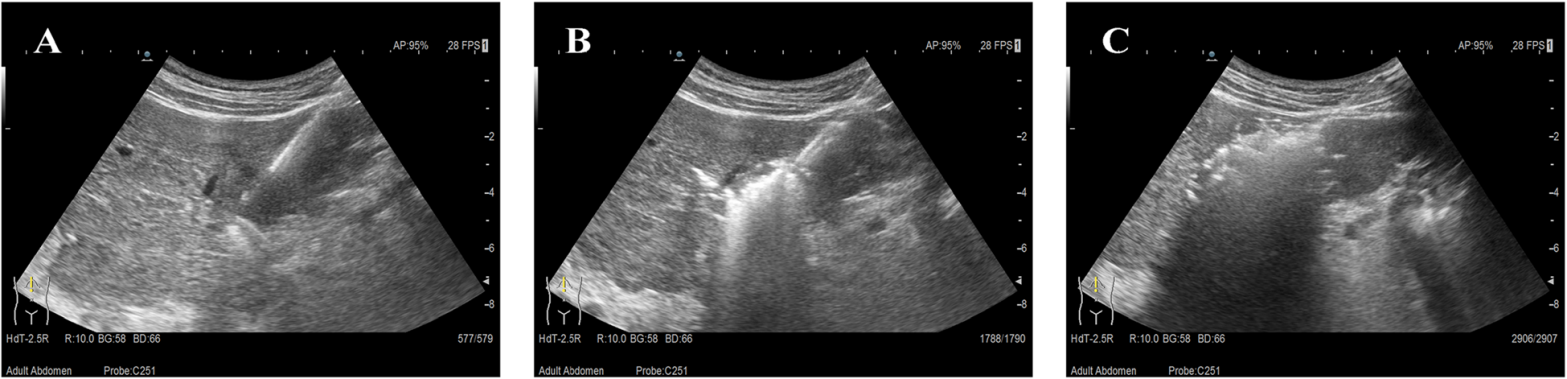
Radiofrequency ablation (RFA) for the treatment of patients with recurrent colorectal liver metastases after hepatectomy. **a** Needles placement. **b** Start of the ablation. **c** Vaporizing tumor

RFA was performed using the Korean starmed cooling cycle therapeutic instrument with a rated output power of 150 W and a 17G unipolar ablation radiofrequency needle (effective ablation radius of 30 mm). Ultrasound-guided interventional therapy was performed by ALOKAα7 ultrasonic diagnostic apparatus with a convex array probe (frequency 2–6 MHz), linear array probe (frequency 4–10 MHz), and laparoscopic ultrasound probe (frequency 4–10 MHz). RFA treatment was done by two experienced ultrasound interventional physicians. The direction and depth of electrode placement were determined in accordance with preoperative and intraoperative ultrasonography. One to three ablation electrodes were placed in the tumor after the puncture needle was put into the central part of the tumor. The tumors were ablated under the setting procedure and protocol. When tumor size was more than 3 cm in diameter, overlapping ablation was adopted according to the shape, size, and location of the tumors. The ablation range reached 0.5–1.0 cm in a way that maintains security and eliminates the tumors and possible infiltration to a great extent. At the end of treatment, the ablation electrode was withdrawn and use needle ablation mode, as shown in Fig. 1.

### Repeat hepatic resection

Of the 50 patients treated with hepatectomy, 27 were single metastases, 14 had two metastatic hepatic nodules, and nine had more than three metastases. In 29 cases, metastases were confined to a single lobe, and 21 involved both left and right lobe. Twenty-eight patients had a diameter of less than 5 cm (the sum of the diameters of multiple lesions), and 22 patients had a diameter of more than 5 cm.

Local enucleation accounted for 76% (38 cases), hepatic segmentectomy 8% (four cases), left lateral hepatectomy 12% (six cases), and left hepatectomy 4% (two cases).

### Clinical detection

The level of ALT, AST, albumin (ALB), and total bilirubin (TBIL) were detected by automatic biochemical analyzer before and the 7th day after treatment.

### Follow-up

Postoperative adjuvant chemotherapy was given based upon the individual difference, disease condition, and biochemical test results. All patients were followed up regularly and the deadline date was January 2019. Serum tumor markers were examined and enhanced CT or MRI was performed to evaluate the local ablation of tumors 1 month postoperatively. The therapeutic effect was analyzed by enhanced CT or MRI every 3 months within 2 years and every 6 months after 2 years. The survival rate was recorded during follow-up. The overall survival (OS) started from the date of initiated therapy, ended in the dead day or the last follow-up date, and DFS was calculated from the day of therapy until the first recurrence or death.

### Statistical analysis

The preoperative clinical data, perioperative results, and survival status of the patients were collected. Statistical analyses were performed using SPSS 22.0. Continuous variables were expressed as mean ± standard deviation (SD) and *t* test was used for comparison between the two groups. Categorical variables were expressed as relative numbers and *χ*^*2*^ test was used for comparison between the two groups. The primary endpoints were DFS and OS, and the survival curves were depicted by the Kaplan-Meier method, with comparisons using the Log-rank test. To overcome the influence of selection bias and confounding factors on the results, propensity score analysis was applied. In addition to therapeutic methods, all variables affecting prognosis were included as covariates in the logistic regression model to estimate the probability that the subjects were assigned to two groups. Individuals with similar probabilities were selected and then matched from the two groups to meet the randomization criteria. In this study, RFA and repeat hepatic resection group were matched as 1:2, and the caliper value was 0.2. The level of significance was set at *P* ≤ 0.05 for all tests.

## Result

### Comparison of clinical data between two groups

A total of 92 lesions were ablated in 50 patients in the repeat hepatic resection group and the average number was (2.7 + 0.8). The maximum diameter of tumors was 4.7 cm, with an average of (3.2 + 1.1) cm. RFA group had an average metastatic lesion of (5.1 + 1.3), and 258 metastatic lesions were eliminated. The overall ablation rate of tumors was 90.7% (234/258) within 1 month after treatment and 12 lesions were partial ablation. The maximum diameter of ablation lesions was 3.6 cm and the average was (2.6 + 0.9) cm.

Fifty-one patients underwent primary ablation, 63 cases needed a repeated ablation, and for 30 cases ablation was carried out three times or more. The local recurrence rate was 7.0% (18/258). Intrahepatic neonatal metastases were found in 114 patients after treatment, the incidence rate was 79.2% (114/144).

Clinical characteristics of tumors removed in 194 patients are summarized in Table 1. Statistical analysis showed comparability that two groups of preoperative chemotherapy, number of metastatic tumors, and distribution of liver metastases were significantly different. The number of metastases in the RFA group was higher than that in the repeat hepatic resection group (*P* = 0.022), and the proportion of left and right lobe involved by tumor was 47.9%, obviously higher than that of repeat hepatic resection (*P* = 0.004). Up to 44.4% of patients in the RFA group received preoperative chemotherapy, while only 30.0% of patients in the repeat hepatic resection group received preoperative chemotherapy.

**Table 1:** Comparison of clinical data between two groups of patients with recurrent colorectal liver metastases after hepatectomy

Variable		RFA (*n* = 144)	Repeat hepatic resection (*n* = 50)	*χ*^2^	*P*	*v*
Sex ratio (*n*)	Male:Female	87:57:00	33:17:00	0.674	0.411	1
Age(years)	≤ 60	66 (45.8)	21 (42.0)	0.293	0.588	1
	> 60	78 (54.2)	29 (58.0)			
Primary tumors location (*n*%)	Colon	90 (62.5)	30 (60.0)	0.132	0.717	1
	Rectum	54 (37.5)	20 (40.0)			
Timing of liver metastases (*n*%)	Synchronous	117 (81.3)	35 (70.0)	3.466	0.063	1
	Metachronous	27 (18.7)	15 (30.0)			
Distribution of liver metastases (*n*%)	Unilobar	75 (52.1)	36 (72.0)	8.409	0.004	1
	Bilobar	69 (47.9)	14 (28.0)			
Preoperative chemotherapy (*n*%)		64 (44.4)	15 (30.0)	4.438	0.035	1
CEA (ng/ml)	≤ 10	87 (60.4)	34 (68.0)	1.257	0.262	1
	> 10	57 (39.6)	16 (32.0)			
Size of tumors (cm)	≤ 3	102 (70.8)	37 (74.0)	0.256	0.613	1
	> 3	42 (29.2)	13 (26.0)			
Number of metastatic tumors (*n*%)	Solitary	63 (43.8)	30 (60.0)	5.256	0.022	1
	Multiple	81 (56.2)	20 (40.0)			
Relapse time interval (months)	≤ 6	57 (39.6)	14 (28.0)	3.007	0.083	1
	> 6	87 (60.4)	36 (72.0)			

Values in parentheses are percentages; *RFA* radiofrequency ablation, *CEA* carcinoembryonic antigen

### Propensity score analysis

According to the maximum diameter of metastases (< 3 cm or > 3 cm), distribution of metastases (unilobar or bilobar), number of metastases (single or multiple), relapse time interval (< 6 months or > 6 months), age (< 60 years or > 60 years), carcinoembryonic antigen (CEA) level (< 10 ng/ml or > 10 ng/ml), time of liver metastasis (synchronous or metachronous), lymph node metastasis (yes or no), and preoperative chemotherapy (yes or no), propensity score analysis was used to analyze the effect of RFA on survival and recurrence.

Using the propensity score matching method, there were 62 cases in the RFA group and 31 cases in the repeat hepatic resection group. The gender, age, primary tumor location, relapse time interval after hepatectomy, metastases distribution, preoperative chemotherapy, extrahepatic metastasis, CEA level, metastases number, and maximum diameter showed no significant difference between two groups (*P* > 0.05), as shown in Table 2.

**Table 2:** Background characteristics of the matched cohorts

Variable		RFA (*n* = 62)	Repeat hepatic resection (*n* = 31)	*χ*^2^	*P*	*v*
Sex ratio (*n*)	Male:Female	41:21:00	18:13	1.36	0.244	1
Age(years)	≤ 60	30 (48.4)	14 (45.2)	0.206	0.65	1
	> 60	32 (51.6)	17(54.8)			
Primary tumor location (*n*%)	Colon	37 (59.7)	19 (61.3)	0.054	0.817	1
	Rectum	25 (40.3)	12 (38.7)			
Timing of liver metastases (*n*%)	Synchronous	47 (75.8)	22 (71.0)	0.59	0.442	1
	Metachronous	15 (24.2)	9 (29.0)			
Distribution of liver metastases (*n*%)	Unilobar	37 (59.7)	20 (64.5)	0.489	0.484	1
	Bilobar	25 (40.3)	11 (35.5)			
Preoperative chemotherapy (*n*%)		24 (38.7)	11 (35.5)	0.219	0.639	1
CEA (ng/ml)	≤ 10	39 (62.9)	20 (64.5)	0.055	0.814	1
	> 10	23 (37.1)	11 (35.5)			
Size of tumors (cm)	≤ 3	45 (72.6)	21 (67.7)	0.573	0.449	1
	> 3	17 (27.4)	10 (32.3)			
Number of metastatic tumors (*n*%)	Solitary	31 (50.0)	17 (54.8)	0.462	0.497	1
	Multiple	31 (50.0)	14 (45.2)			
Relapse time interval (months)	≤ 6	22 (35.5)	10 (32.2)	0.238	0.625	1
	> 6	40 (64.5)	21 (67.7)			

Values in parentheses are percentages; RFA: radiofrequency ablation; CEA: carcinoembryonic antigen.

### Comparisons of liver function-related indexes between two groups before and after treatment

ALT and AST levels in both groups were significantly higher than those before treatment, while ALB levels were lower than those before treatment on the 7th day after treatment, and the difference was statistically significant (*P* < 0.05). Although TBIL increased to some extent after treatment, the difference was not statistically significant (*P* > 0.05). Compared with the RFA group, the liver function of the repeat hepatic resection group was significantly improved. However, no significant difference in liver function-related indexes was found between RFA and repeat hepatic resection after matching (Table 3).

**Table 3:** Comparison of liver function indexes between two groups before and after treatment

	Preoperation				Postoperation			
	ALT (U/L)	AST (U/L)	ALB (g/L)	TBIL (μmol/L)	ALT (U/L)	AST (U/L)	ALB (g/L)	TBIL (μmol/L)
Whole cohort								
RFA (*n* = 144)	42.3 ± 17.5	41.8 ± 18.4	43.4 ± 4.6	21.7 ± 9.2	68.1 ± 32.4^*^	63.6 ± 24.9^*^	38.5 ± 3.9^*^	23.2 ± 7.3
Repeat hepatic resection (*n* = 50)	41.2 ± 15.2	40.6 ± 22.3	42.7 ± 3.8	19.6 ± 11.1	61.2 ± 35.8^*#^	57.7 ± 28.3^*#^	40.4 ± 2.5^*^	22.1 ± 8.6
Matched cohort								
RFA (*n* = 62)	39.7 ± 20.8	38.3 ± 20.5	44.1 ± 3.7	18.2 ± 10.6	56.6 ± 24.3^*^	55.0 ± 29.4^*^	41.8 ± 2.6^*^	20.7 ± 11.1
Repeat hepatic resection (*n* = 31)	40.9 ± 24.0	41.2 ± 25.6	43.6 ± 3.2	19.4 ± 9.5	55.7 ± 30.5^*^	58.1 ± 26.2^*^	40.5 ± 3.1^*^	20.4 ± 10.9

Compare with preoperation: ^*^P < 0.05; compare with RFA: ^#^*P* < 0.05; whole cohort: *v* = 192; matched cohort: *v* = 30

*RFA* radiofrequency ablation, *ALB* albumin, *TBIL* total bilirubin

### Postoperative complications

No perioperative death or acute liver failure occurred in both groups after treatment, and all patients were discharged successfully. In repeat hepatic resection group, 16 patients had postoperative complications, including hemorrhage in three cases, incision infection in four cases, abdominal infection in three cases, bile leakage in three cases, gastrointestinal injury in two cases, and hydrops abdominis in one case. In the RFA group, there were three cases of gastrointestinal injury, four cases of diaphragm injury, three cases of intraperitoneal hemorrhage, five cases of infection, two cases of pleural effusion, and seven cases of nausea, vomiting, and upper epigastric discomfort. Comparison of two parameters of the complications, the result shown that the incidence of complications in the RFA group was lower than that in the repeat hepatic resection group (*P* = 0.037).

After using the propensity score, as shown in Table 4, the operation time and postoperative hospital stay in the RFA group were significantly lower than those in the repeat hepatic resection group. Postoperative complications were mainly hemorrhage, infection, and biliary leakage. Patients who had undergone RFA showed a significant lower in complications incidence as compared with repeat hepatic resection, and the difference was statistically significant (*P* < 0.05).

**Table 4:** Comparison of intraoperative and postoperative outcomes of the matched cohorts

Variable	RFA (*n* = 62)	Repeat HEPATIC Resection (*n* = 31)	*P*	*v*
Operation time (min)	106.9 ± 41.4	232.5 ± 68.3	0.000	30
Postoperative hospital stay (days)	10.6 ± 5.8	14.5 ± 7.2	0.006	30
Complications (*n*%)			0.544	7
Hemorrhage	1	2		
Bile leakage	0	2		
Infection	3	3		
Gastrointestinal burns	3	1		
Diaphragm burns	2	0		
Hepatic arteriovenous fistula	0	1		
Abdominal infection	2	2		
Hepatic failure	0	0		

*RFA* radiofrequency ablation

### Analysis of overall survival time and recurrence

**Fig. 2:**
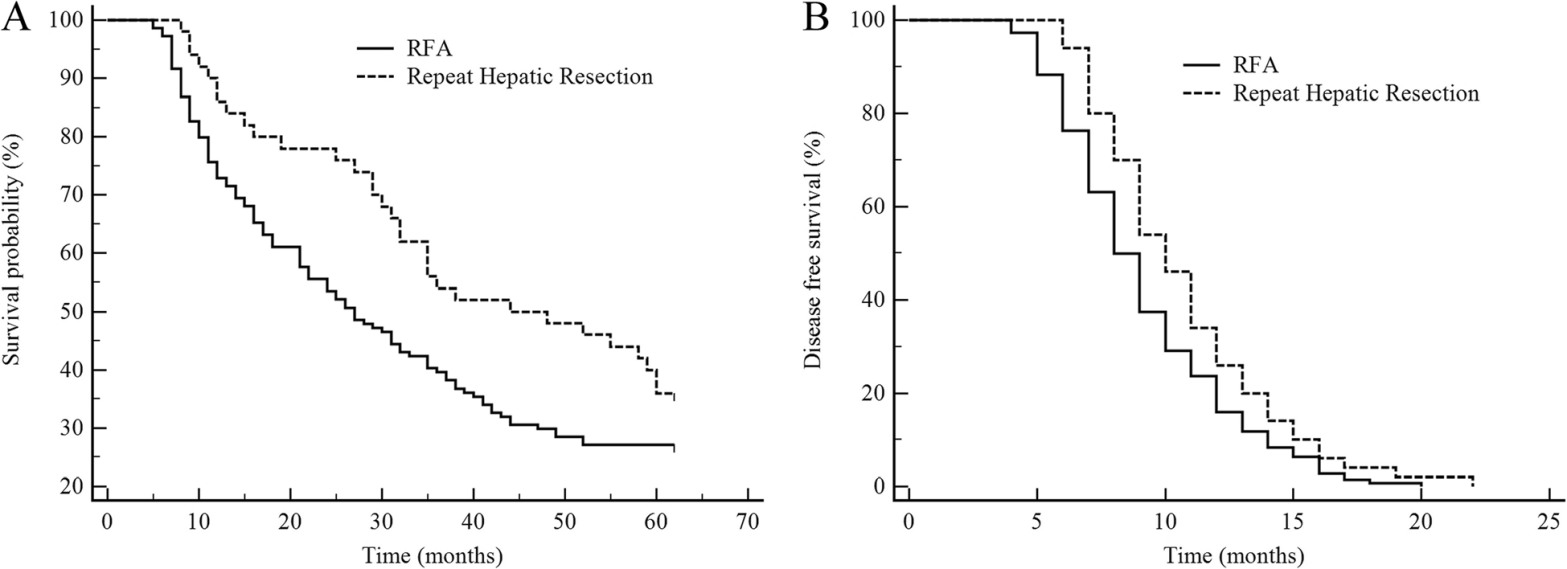
Survival comparisons in patients who underwent radiofrequency ablation (RFA) and repeat hepatic resection before matching. **a** Overall survival (OS). **b** Disease-free survival (DSF)

By the end of January, 2019, the median follow-up period of the full cohort of 194 patients with recurrent colorectal liver metastases was 28.6 months, of whom 67 (52.8%) were survival and 127 (65.5%) dead. The 1, 2, and 3 years OS rates were 83.0%, 53.1%, and 34.5% respectively. Figure 2a showed that the 5-year OS rates of repeat hepatic resection group and RFA group were 36.0% (32) and 27.1% (105), respectively. Compared with the RFA group, the reoperation group had a higher OS rate, indicating a better prognosis (*χ*^*2*^ = 4.024, *P* = 0.045). After treatment, the median DFS of the RFA group was 7.2 months, shorter than 11.3 months of repeat hepatic resection group (*χ*^*2*^ = 5.425, *P* = 0.002), as shown in Fig. 2b.

**Fig. 3:**
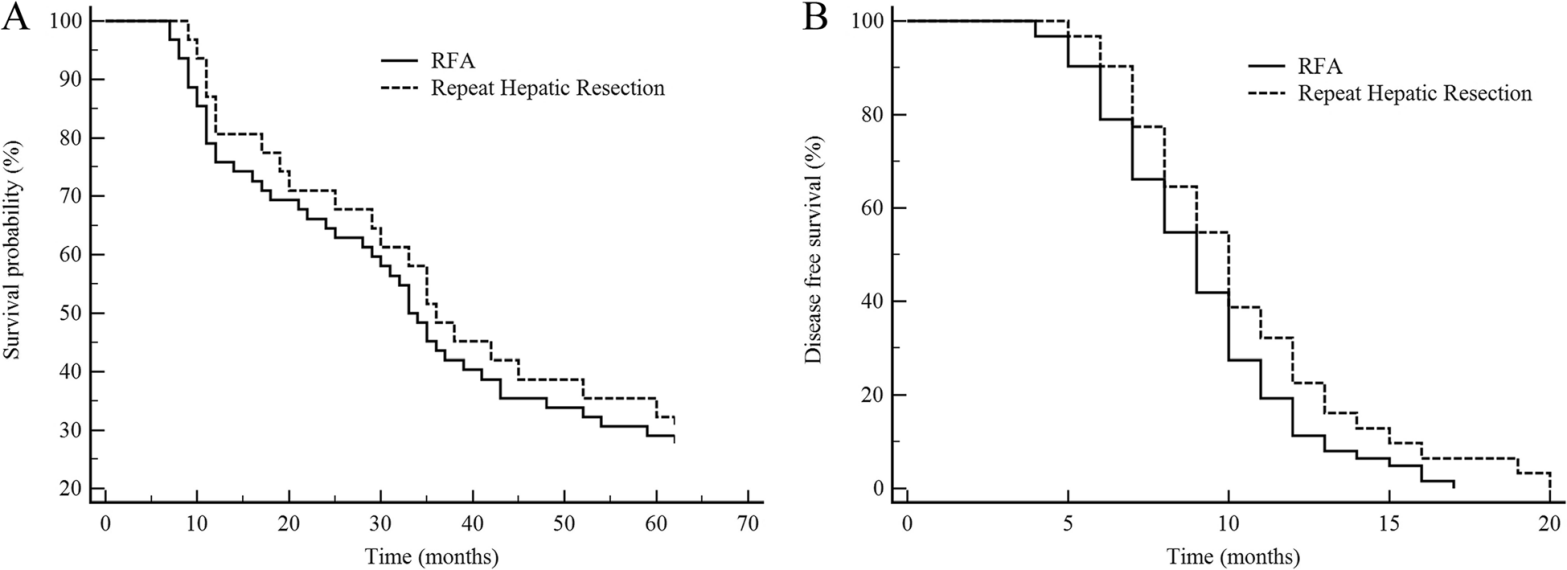
Survival comparisons: radiofrequency ablation (RFA) versus repeat hepatic resection after matching. **a** Overall survival (OS). **b** Disease-free survival (DSF)

After propensity score matching, the result showed that the median survival time of 31 patients treated with repeat hepatic resection was 36.7 months, and the OS rates of 1, 3, and 5 years were 80.6% (6), 48.4% (16), and 32.3% (21), respectively. The median survival time of 62 patients who undergo RFA was 31.9 months, and the OS rates of 1, 3, and 5 years were 75.8% (15), 43.5% (35), and 29.0% (44), respectively. There was no significant difference in OS and DFS between the two groups, as shown in Fig. 3a, b.

## Discussion

The survival time and quality of life of colorectal cancer patients with liver metastasis are determined by the therapeutic effect of metastases. Hepatectomy is the optimal treatment option for individuals, but the high rate of postoperative recurrence and metastasis has gradually become a problem [25, 26]. Relevant reports confirmed that tumor recurrence is detected in 60–70% of patients with liver metastasis from colorectal cancer following curative resection, and 30% of them were often found in the liver [27]. Subsequent research has found that repeat hepatic resection for recurrent patients could get an ideal therapeutic effect. Therefore, repeat hepatic resection can be used as a favorable choice of treating recurrent patients. It can be applied repeatedly in patients without surgical contraindication [16, 28, 29]. Nevertheless, the risk of repeat hepatic resection is higher than that of initial surgery. Only a few patients can meet treatment criteria, while most patients are candidates for non-surgical treatment, among which the most commonly used is RFA [30, 31]. RFA, characterized by well-tolerated and easily repeated, has been widely used in the minimally invasive treatment of liver cancer. However, the long-term effect of RFA is not definite compared with the repeat hepatic resection [32].

Traditionally, RFA has been used to treat patients who were not candidates for surgical resection, such as extrahepatic metastases, serious primary disease, and special tumor location, to improve their postoperative survival rate [33, 34]. Previous studies have reported that the survival rate of patients with RFA was only moderately higher than other non-surgical treatments. Besides, extrahepatic thermal injury and needle track implantation are more likely to occur in patients undergoing RFA, which cannot achieve the same effect as surgical resection [35]. Thereby, RFA was recommended as a good supplement for recurrent colorectal liver metastases after hepatectomy. Recent evidence indicates that the poor prognosis of patients was mainly related to aggressive tumor behavior. Patients with multiple metastases and large diameters have a significantly worse prognosis than that of patients with less tumor burden [36, 37], which suggests that the negative effect of RFA on the prognosis of patients may be caused by aggressive tumor biological behavior. While patients with the superficial tumors or better liver function are commonly treated by repeat hepatic resection, resulting in a significant difference in baseline data between the two groups. In this study, patients treated with RFA also showed more serious disease states. Compared with the repeat hepatic resection group, the number of metastases, the proportion of left and right lobe involved, and preoperative chemotherapy in RFA were higher. To reduce bias between two compared groups, propensity score analysis was used. On this basis, the effects of treatment factors were further evaluated. After adjustment for potential confounders, the clinical data of the two groups were not statistically significant.

Alterations in AST and ALT are very sensitive following liver function damage and can reflect the degree of damage. Both TBIL and ALB are closely related to the synthetic function of the liver and can reflect the liver reserve function. Seven days after surgery, the ALT and AST levels of the two groups were significantly higher than before treatment, while the ABL was significantly lower than before treatment, which suggests that the liver had a certain degree of damage. Whether it is repeat hepatic resection or RFA, normal liver tissues are mostly involved when tumor cells are eliminated, leading to transient damage of liver function. Thus, preoperative liver function assessment and postoperative liver protection treatment are particularly important for patients with recurrent colorectal liver metastasis. Furthermore, we evaluated the damage severity of liver tissues in both groups. The results showed that the degree of liver injury in the repeat hepatic resection group was less than that in the RFA group without adjustment on major confounding factors and there was no statistical difference in liver function-related indexes between the two groups after adjusting. Additionally, we analyzed the incidence and type of complications in the two groups. Compared with the repeat hepatic resection group, the RFA group has fewer complications, mainly including intraperitoneal hemorrhage, fever, abdominal pain, poor appetite, gastrointestinal injury, bile duct injury, bile leakage, pleural effusion, pin tract infection, and needle track implantation. The incidence of serious complications was less than 3.0%. The advantage of RFA in the treatment of patients is that it can reduce the damage to normal liver tissue and important blood vessels, and the operation time and postoperative hospital time are significantly lower than those in the repeat hepatic resection group. This shows that RFA has the same short-term effect on patients as the repeat hepatic resection under the condition of similar tumor load.

A two-center prospective study found that the 1-, 3-, and 5-year survival rates were 86.0%, 51.0%, and 34% in patients who received repeat hepatic resection, similar to those in patients without recurrence after primary hepatectomy [38]. In this study, the survival rate of patients who underwent the second resection was 36.0%, which was consistent with the above research. Compared with the 5-year survival rate of 20~58% in the first surgery, there was no significant difference between the two operations. Repeat hepatic resection provides a cure opportunity for some recurrent patients with mild clinical symptoms, which will benefit these patients obviously. Another study showed that the DFS of primary, second, and third operation in patients with recurrent colorectal liver metastases after hepatectomy were 1.3 years, 1.1 years, and 2.0 years, respectively, which was similar to the results of this study. In our study, the 5-year OS and DFS of RFA group were lower than those of repeat hepatic resection group, but the two groups obtained similar overall prognosis results after using propensity score, indicating that there was no significant difference in the long-term effect between RFA group and repeat hepatic resection group.

The inadequacies of this study mainly display in the following aspects: (1) although propensity scores were used, we failed to achieve complete randomization. (2) This study sample size was small. (3) Recurrent colorectal liver metastases are a systemic disease, and patients need to receive systemic chemotherapy (FOLFOX/FOLFIRI) before and after treatment. Chemotherapy usually lasts 6 months, with a cycle of 20 days. However, due to the severity and progression of the disease, there are differences in the postoperative chemotherapy time. The choice and duration of chemotherapy are also important factors affecting the prognosis of the patient. Therefore, large sample, prospective, randomized controlled studies on treatment are expected to confirm the safety and clinical effect of RFA.

## Conclusion

RFA is safe and feasible for patients with recurrent colorectal liver metastases after hepatectomy, which has long-term efficacy comparable to that of repeat hepatic resection and fewer complications.

## Data Availability

The datasets during the current study are available from the corresponding author on reasonable request.

## Abbreviations

RFA: Radiofrequency ablation
CEA: Carcinoembryonic antigen
ALB: Albumin
TBIL: Total bilirubin
OS: Overall survival
DSF: Disease-free survival

## References

[1] Engstrand J, Nilsson H, Strömberg C, Jonas E, Freedman J (2018). Colorectal cancer liver metastases—a population-based study on incidence, management and survival. BMC Cancer..

[2] Al Bandar MH, Kim NK (2017). Current status and future perspectives on treatment of liver metastasis in colorectal cancer (Review). Oncol Rep..

[3] Lee BC, Lee HG, Park IJ, Kim SY, Kim KH, Lee JH, Kim CW, Lee JL, Yoon YS, Lim SB (2016). The role of radiofrequency ablation for treatment of metachronous isolated hepatic metastasis from colorectal cancer. Medicine (Baltimore).

[4] Smith JJ, D'Angelica MI (2015). Surgical management of hepatic metastases of colorectal cancer. Hematol Oncol Clin North Am..

[5] Toso C, Pinto Marques H, Andres A, Castro Sousa F, Adam R, Kalil A, Clavien PA, Furtado E, Barroso E, Bismuth H (2017). Compagnons Hépato-Biliaires Group. Liver transplantation for colorectal liver metastasis: Survival without recurrence can be achieved. Liver Transpl..

[6] Engstrand J, Kartalis N, Strömberg C, Broberg M, Stillström A, Lekberg T, Jonas E, Freedman J, Nilsson H (2017). The impact of a hepatobiliary multidisciplinary team assessment in patients with colorectal cancer liver metastases: a population-based study. Oncologist..

[7] Fukuoka K, Nara S, Honma Y, Kishi Y, Esaki M, Shimada K (2017). Hepatectomy for colorectal cancer liver metastases in the era of modern preoperative chemotherapy: evaluation of postoperative complications. World J Surg..

[8] Hodgson R, Sethi H, Ling AH, Lodge P (2017). Combined hepatectomy and hepatic pedicle lymphadenectomy in colorectal liver metastases is justified. HPB (Oxford).

[9] Folprecht G (2016). Liver Metastases in Colorectal Cancer. Am Soc Clin Oncol Educ Book..

[10] Dhar V, Thomas RM, Ahmad SA (2016). Repeat hepatectomy for colorectal liver metastases. Cancer Treat Res..

[11] Snoeren N, van Hooff SR, Adam R, van Hillegersberg R, Voest EE, Guettier C, van Diest PJ, Nijkamp MW, Brok MO, van Leenen D (2012). Exploring gene expression signatures for predicting disease free survival after resection of colorectal cancer liver metastases. PLoS One..

[12] Akgül Ö, Çetinkaya E, Ersöz Ş, Tez M (2014). Role of surgery in colorectal cancer liver metastases. World J Gastroenterol..

[13] Konopke R, Volk A, Gastmeier J, Ehehalt F, Distler M, Saeger HD, Kersting S (2014). Recurrent colorectal liver metastases: who benefits from a second hepatic resection?. Zentralbl Chir..

[14] Dupré A, Rehman A, Jones RP, Parker A, Diaz-Nieto R, Fenwick SW, Poston GJ, Malik HZ (2018). Validation of clinical prognostic scores for patients treated with curative-intent for recurrent colorectal liver metastases. J Surg Oncol..

[15] Viganò L, Lopci E, Costa G, Rodari M, Poretti D, Pedicini V, Solbiati L, Chiti A, Torzilli G (2017). Positron emission tomography-computed tomography for patients with recurrent colorectal liver metastases: impact on restaging and treatment planning. Ann Surg Oncol..

[16] Shaw IM, Rees M, Welsh FK, Bygrave S, John TG (2006). Repeat hepatic resection for recurrent colorectal liver metastases is associated with favourable long-term survival. Br J Surg..

[17] Lee H, Choi SH, Cho YB, Yun SH, Kim HC, Lee WY, Heo JS, Choi DW, Jung KU, Chun HK (2015). Repeat hepatic resection in patients with colorectal liver metastases. World J Gastroenterol..

[18] Nanji S, Tsang ME, Wei X, Booth CM (2017). Outcomes after repeat hepatic resection for recurrent metastatic colorectal cancer: a population-based study. Am J Surg..

[19] Kulik U, Bektas H, Klempnauer J, Lehner F (2013). Repeat liver resection for colorectal metastases. Br J Surg..

[20] Neeff HP, Drognitz O, Holzner P, Klock A, Bronsert P, Hopt UT, Makowiec F (2013). Outcome after repeat resection of liver metastases from colorectal cancer. Int J Colorectal Dis..

[21] van Amerongen MJ, Jenniskens SFM, van den Boezem PB, Fütterer JJ, de Wilt JHW (2017). Radiofrequency ablation compared to surgical resection for curative treatment of patients with colorectal liver metastases—a meta-analysis [J]. HPB (Oxford).

[22] Cirimbei C, Rotaru V, Chitoran E, Pavaleanu O, Cirimbei SE (2017). Immediate and Long-term Results of Radiofrequency Ablation for Colorectal Liver Metastases [J]. Anticancer Res..

[23] Facciorusso A, Del Prete V, Muscatiello N, Crucinio N, Barone M (2016). Prognostic role of 25-hydroxyvitamin D in patients with liver metastases from colorectal cancer treated with radiofrequency ablation. J Gastroenterol Hepatol..

[24] Hof J, Joosten HJ, Havenga K, de Jong KP (2018). Radiofrequency ablation is beneficial in simultaneous treatment of synchronous liver metastases and primary colorectal cancer. PLoS One..

[25] Watanabe A, Araki K, Harimoto N, Kubo N, Igarashi T, Ishii N, Yamanaka T, Hagiwara K, Kuwano H, Shirabe K (2018). D-dimer predicts postoperative recurrence and prognosis in patients with liver metastasis of colorectal cancer. Int J Clin Oncol..

[26] Yamaguchi S, Yokoyama S, Ueno M, Hayami S, Mitani Y, Takeuchi A, Shively JE, Yamaue H (2017). CEACAM1 is associated with recurrence after hepatectomy for colorectal liver metastasis. J Surg Res..

[27] Pandanaboyana S, Bell R, White A, Pathak S, Hidalgo E, Lodge P, Prasad R, Toogood G (2018). Impact of parenchymal preserving surgery on survival and recurrence after liver resection for colorectal liver metastasis. ANZ J Surg..

[28] Araujo RL, Gönen M, Allen P, DeMatteo R, Kingham P, Jarnagin W, D'Angelica M, Fong Y (2015). Positive postoperative CEA is a strong predictor of recurrence for patients after resection for colorectal liver metastases. Ann Surg Oncol..

[29] Imai K, Allard MA, Benitez CC, Vibert E, Sa Cunha A, Cherqui D, Castaing D, Bismuth H, Baba H, Adam R (2016). Early recurrence after hepatectomy for colorectal liver metastases: what optimal definition and what predictive factors [J]. Oncologist..

[30] Takahashi M, Hasegawa K, Oba M, Aoki T, Sakamoto Y, Sugawara Y, Kokudo N (2015). Repeat resection leads to long-term survival: analysis of 10-year follow-up of patients with colorectal liver metastases. Am J Surg..

[31] de Haas RJ, Wicherts DA, Salloum C, Andreani P, Sotirov D, Adam R, Castaing D, Azoulay D (2010). Long-term outcomes after hepatic resection for colorectal metastases in young patients. Cancer..

[32] Hashimoto M, Kobayashi T, Ishiyama K, Ide K, Ohira M, Tahara H, Kuroda S, Hamaoka M, Iwako H, Okimoto M (2016). Efficacy of repeat hepatectomy for recurrence following curative hepatectomy for colorectal liver metastases: a Retrospective Cohort Study of 128 patients. Int J Surg..

[33] Xu C, Huang XE, Lv PH, Wang SX, Sun L, Wang FA (2015). Radiofrequency Ablation in Treating Colorectal Cancer Patients with Liver Metastases. Asian Pac J Cancer Prev..

[34] Nielsen K, van Tilborg AA, Meijerink MR, Macintosh MO, Zonderhuis BM, de Lange ES, Comans EF, Meijer S, van den Tol MP (2013). Incidence and treatment of local site recurrences following RFA of colorectal liver metastases. World J Surg..

[35] Kim WW, Kim KH, Kim SH, Kim JS, Park SJ, Kim KH, Choi CS, Choi YK (2015). Comparison of hepatic resection and radiofrequency ablation for the treatment of colorectal liver metastasis. Indian J Surg..

[36] Sasaki K, Margonis GA, Andreatos N, Kim Y, Wilson A, Gani F, Amini N, Pawlik TM (2016). Combined resection and RFA in colorectal liver metastases: stratification of long-term outcomes. J Surg Res..

[37] He N, Jin QN, Wang D, Yang YM, Liu YL, Wang GB, Tao KX (2016). Radiofrequency ablation vs. hepatic resection for resectable colorectal liver metastases. J Huazhong Univ Sci Technolog Med Sci..

[38] Heise D, Bayings W, Tuinhof A, Eickhoff R, Kroh A, Ulmer F, Dejong CHC, Neumann U, Binnebösel M (2017). Long-term outcome and quality of life after initial and repeat resection of colorectal liver metastasis: a retrospective analysis. Int J Surg..

